# An ever-changing landscape in Roberts syndrome biology: Implications for macromolecular damage

**DOI:** 10.1371/journal.pgen.1009219

**Published:** 2020-12-31

**Authors:** Michael G. Mfarej, Robert V. Skibbens

**Affiliations:** Department of Biological Sciences, Lehigh University, Bethlehem, Pennsylvania, United States of America; Geisel School of Medicine at Dartmouth, UNITED STATES

## Abstract

Roberts syndrome (RBS) is a rare developmental disorder that can include craniofacial abnormalities, limb malformations, missing digits, intellectual disabilities, stillbirth, and early mortality. The genetic basis for RBS is linked to autosomal recessive loss-of-function mutation of the establishment of cohesion (ESCO) 2 acetyltransferase. *ESCO2* is an essential gene that targets the DNA-binding cohesin complex. ESCO2 acetylates alternate subunits of cohesin to orchestrate vital cellular processes that include sister chromatid cohesion, chromosome condensation, transcription, and DNA repair. Although significant advances were made over the last 20 years in our understanding of ESCO2 and cohesin biology, the molecular etiology of RBS remains ambiguous. In this review, we highlight current models of RBS and reflect on data that suggests a novel role for macromolecular damage in the molecular etiology of RBS.

## Introduction

Roberts syndrome (RBS) (MOM #268300, MIM#269000) is a severe developmental disorder, first described in 1919, in which patients exhibit prenatal growth retardation, limb malformations, and craniofacial abnormalities [1–7]. Mildly affected individuals can survive to adulthood, but severely affected cases result in spontaneous abortion, stillbirth, or death within 1 month [5,6]. The treatment of RBS is limited to prevention, surgery to correct physical malformations, prostheses, special education, speech therapy, and treatments for organ dysfunction [5,6].

**Fig 1:**
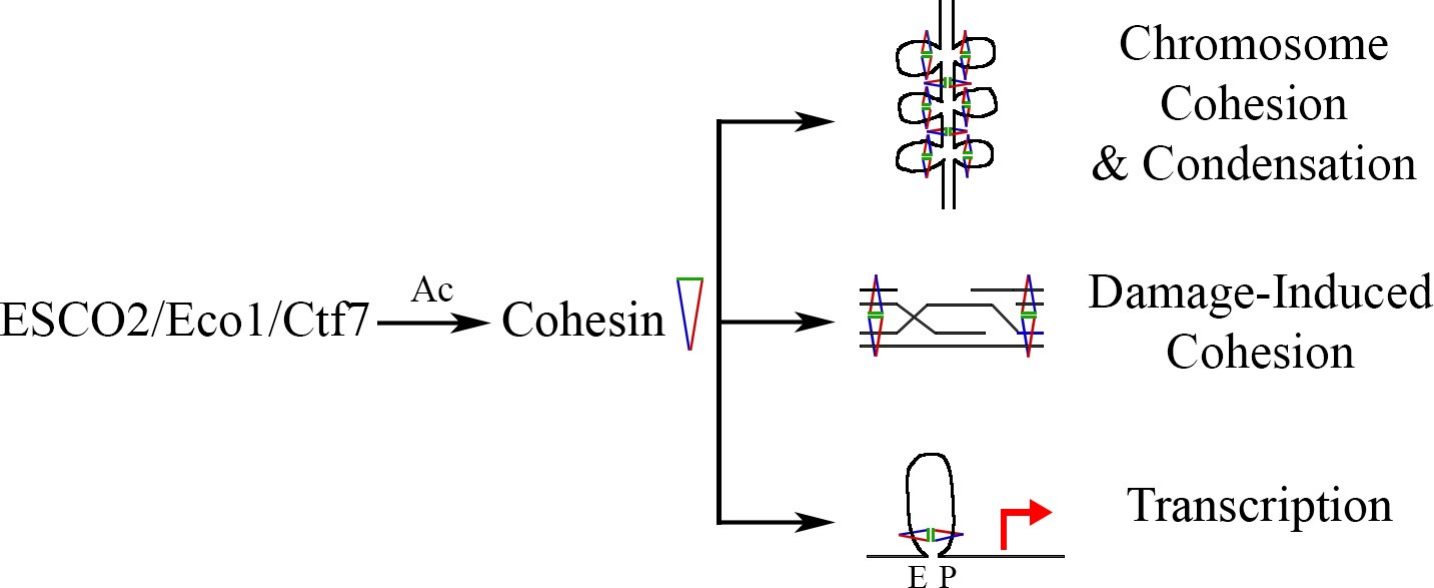
ESCO2 regulates genome structure, function, and stability. ESCO2 (Eco1/Ctf7 in yeast) is an acetyltransferase that modifies cohesin proteins during chromosome cohesion and condensation. Cohesin acetylation drives DNA damage-induced cohesion, which brings sister chromatids into close physical proximity to promote strand invasion during homologous recombination. ESCO2 acetylates cohesin to regulate chromatin looping, thereby modulating transcriptional outputs that bring enhancers (E) and promoters (P) into close proximity. Ac, acetylation; Ctf7, chromosome transmission fidelity 7 protein; Ecol, establishment of cohesion 1 protein; ESCO2, establishment of cohesion 2 protein.

Surprisingly, autosomal recessive loss-of-function mutation in establishment of cohesion (*ESCO2*) 2 (*ECO1/CTF7* in yeast), which encodes an essential acetyltransferase, is the sole genetic cause for the profound birth defects observed in RBS patients [8–10]. ESCO2 acetylates various components of the cohesin complex that in turn ensures genomic stability [11–14]. Cohesin is a multi-subunit protein, which in humans is composed of structural maintenance of chromosomes protein 1A (SMC1A), structural maintenance of chromosomes protein 3 (SMC3), radiation-sensitive 21 homolog protein (RAD21), stromal antigen proteins 1 and 2 (SA1/2) (Smc1, Smc3, Mcd1/Scc1, and Scc3/Irr1 in yeast, respectively), and auxiliary factors precocious dissociation of sisters proteins 5A and 5B (PDS5A/B) (Pds5 in yeast) and sororin [15–17]. Mutation of cohesin and cohesin regulator genes results in a similarly severe developmental malady termed Cornelia de Lange syndrome [18–23]. Cohesin features a central lumen, which entraps double-stranded DNA and promotes DNA–DNA interactions [24–26]. ESCO2 acetylation of SMC3 promotes sister chromatid cohesion (SCC), chromosome condensation, and transcription, while the acetylation of RAD21 promotes homologous recombination (HR) during DNA repair (Fig 1) [11–14,27–33]. Acetylation of either cohesin subunits stabilizes cohesin binding to DNA such that non-acetylated cohesin is susceptible to the cohesin DNA release factor wings apart-like protein homolog (WAPL) in humans (Wpl1/Rad61 in yeast) [34–36]. Despite the 15-year interval since the identification of *ESCO2* mutation as resulting in RBS, the molecular etiology of RBS is not yet fully understood. Here, current models of RBS are reviewed, and a novel model of macromolecular damage as an underlying factor in RBS is proposed.

### Current models of RBS

Models regarding the molecular defects that underlie RBS continue to evolve. Establishment of cohesion 1 protein, independently termed chromosome transmission fidelity 7 protein (Eco1/Ctf7) was first identified on the basis of chromosome segregation defects [28,29]. *ESCO2* mutation in RBS cells, or depletion in mice, zebrafish, or medaka (Japanese rice fish), all produce mitotic failure (cohesion defects) and elevated rates of apoptosis [10,33,37–39]. Thus, an early and still prevalent model of RBS is one of proliferative stem cell, and developing tissue, loss due to mitotic failure and apoptosis. An issue with this model is that decreasing either Smc3 acetylation or protein levels exerts little impact on SCC. In contrast, these decreases significantly impact condensation, transcription, and DNA repair—only a near-complete absence of Smc3 acetylation abrogates SCC [32,40,41].

An emerging model of RBS is that ESCO2 is a critical regulator of gene expression such that *ESCO2* mutation results in transcriptional misexpression of developmental genes, similar to those that occur in response to cohesin mutations [18,33,42–51]. For instance, zebrafish embryos depleted of ESCO2 exhibit gene dysregulations that overlap with those that result from *rad21* mutation [33,52]. Moreover, morpholino (MO)-directed knockdown (KD) of either ESCO2 or Smc3 reduces bone regeneration in regenerating zebrafish caudal fins, in the absence of increased apoptosis. Instead, bone segment and tissue regeneration defects result, in part, from reduced expression of the gap junction channel protein Cx43. *cx43* mutations result in oculodentodigital dysplasia in humans and bone growth defects in both zebrafish and mice [53–56]. It is tempting to speculate that ESCO2/cohesin directly regulates *cx43* transcription given that (1) exogenous *cx43* expression partially rescues the bone segment growth defects that otherwise result from ESCO2 or Smc3 KD; and (2) Smc3 binds multiple domains upstream of the *cx43* coding sequence [56]. Thus ESCO2, through cohesin, likely regulates enhancer–promoter interactions to modulate gene expression during both bone regeneration and embryo growth.

ESCO2-dependent regulation of gene expression is complex and appears to also involve cohesin-independent modes. Microarray of zebrafish embryos depleted of either cohesin or ESCO2 reveal not only overlapping, but also nonoverlapping gene dysregulations [33,52]. One way that ESCO2 may regulate gene transcription, independent of cohesin, is by exploiting physical interactions with other transcriptional regulators. Interestingly, numerous reports indicate that human ESCO2 binds histone methyltransferases, subunits of the RE1 silencing transcription factor/neural-restrictive silencing cofactor (CoREST) complex that is involved in neuronal gene regulation, and also the Notch transcriptional regulator [57–59]. These findings suggest that ESCO2 can act as a scaffold, which, in association with various transcriptional regulators, represses gene expression in vertebrate cells in a manner that is independent of cohesin. In yeast, Eco1 also appears to regulate a large subset of genes, independent of cohesin activation. For instance, yeast cells that harbor an *eco1-W216G* mutation (analogous to the W539G ESCO2 mutation that results in RBS) exhibit altered expression of 1,210 genes [60]. While *eco1-W216G* mutant yeast cells also exhibit chromosome segregation defects, further evidence supports a role in transcription regulation that may be independent of cohesin. For instance, deletion of *RAD61* bypasses the essential role of Smc3 acetylation by Eco1 [35,36]. Notably, 843 of the 1,210 genes dysregulated in an *eco1* mutant remain dysregulated in *eco1 rad61* double mutant cells [60]. This suggests that a significant subset of gene expressions regulated by Eco1 occur in a manner that is refractory to the cohesin-releasing activity of Rad61 and thus likely independent of cohesin.

Further insights into defective mechanisms that may contribute to RBS appear in the nucleolus. *eco1-W216G* yeast cells also exhibit abnormal nucleoli size and extensive disruption of chromatin organization [60–62]. Immortalized RBS cell lines similarly exhibit markedly fragmented nucleoli [63], revealing that the Eco1/ESCO2 family role in nucleolar function is conserved across evolution. Notably, ESCO2 localizes to nucleoli in mammalian cells [64,65]. Even a brief inactivation of cohesin during G1 impacts the transcription of ribosome maturation genes, and thus nucleolar function, in yeast [66]. The role of ESCO2 in the nucleolus thus constitutes an interesting area of future research that is likely to shed additional light on mechanisms that underlie RBS.

The link between ESCO2-dependent gene transcription and nucleolar structure provides key insights into the translational deficiencies that occur in RBS. To this end, Gerton and colleagues pioneered a compelling body of work that establishes translational dysfunction as a downstream contributor to RBS. Beyond cohesion loss, *eco1-W216G* mutation in yeast also results in impaired translation, which coincides with induction of the stress response transcriptional regulator Gcn4 [60,67]. These translational defects likely arise through defects in both ribosomal DNA (rDNA) transcription and ribosomal maturation [66,67]. Metabolic labeling analysis and altered ribosome profiling in RBS cells confirm that reduced translation is a hallmark of RBS [63,68]. Importantly, stimulation of the mammalian target of rapamycin (mTOR) stress response pathway suppresses both the severity of birth defects in a zebrafish RBS model and also translation deficiencies in RBS fibroblasts [63,68]. These findings suggest that reduced rRNA production and faulty ribosome biogenesis lead to translational defects that promote RBS phenotypes.

### Emerging themes in RBS—Macromolecular damage

#### RBS patient phenotypes as it relates to DNA damage

Although abnormal gene expression and increased cell death are hallmarks of RBS, RBS patient characteristics also are intriguingly reminiscent of recognized diseases of deficient DNA repair. For instance, ataxia–telangiectasia (AT) occurs through autosomal recessive mutation of the ataxia–telangiectasia mutated (ATM) DNA repair signaling kinase and leads to cleft lip and palate and growth retardation [69]. Bloom syndrome (BS) is caused by autosomal recessive mutations in the recombination Q helicase homolog (RECQ) and leads to growth retardation [70]. Fanconi anemia (FA) is linked to the autosomal recessive mutation of several genes involved in HR that result in microcephaly and missing radii and thumbs (https://omim.org). Additionally, Cockayne syndrome (CS) is caused by autosomal recessive mutations in the ERCC6/ERCC8 DNA excision repair factors that lead to growth retardation and microcephaly (https://omim.org). The observation that RBS shares clinical symptoms with diseases of defective DNA repair raises the possibility that the inability to repair DNA damage, which normally arises as a natural byproduct of DNA metabolism (sister chromatid exchanges, replication fork stalling, etc.), is an underappreciated, but important, aspect of the molecular etiology of RBS.

A link between RBS and DNA damage repair-deficient syndromes is evident from numerous studies. RBS patient cells exhibit hypersensitivities to a broad range of genotoxic agents that include the DNA cross-linker mitomycin C (MMC), ionizing radiation (IR), the topoisomerase II inhibitors etoposide, and the topoisomerase I inhibitor camptothecin (CPT) [5,10,64,71–74]. The role for cohesin in DNA damage responses includes both serving as a direct phosphorylation target of checkpoint kinases and promoting DNA repair [30,31,75–80]. This raises the possibility that RBS cell sensitivity to genotoxic stress could be attributed to either a failure to respond to DNA damage and/or a failure to repair the damaged DNA. On the one hand, RBS cell lines exhibit phosphorylation of ATM, checkpoint kinase 1 homolog (Chk1), and the tumor suppressor protein (p53) in response to DNA damaging agents [64,74], suggesting that these checkpoints are functional in RBS cells. On the other hand, several lines of evidence suggest that RBS cells exhibit a reduced ability to repair DNA damage after checkpoint activation. A combination of western blot analyses of whole cell extracts from RBS patient-derived fibroblasts and “Comet” assays shows increased phosphorylated histone 2A variant X (γ-H2AX) levels and chromosomal fragmentation, respectively, that persist long after IR treatment. These results indicate that double-strand breaks (DSBs) persist into subsequent cell cycles due to inefficient repair [74], which likely contributes to increased apoptosis levels. Similarly, immortalized RBS fibroblasts, 24 h after IR or MMC exposure, contain a decreased number of repair recombinase Rad51 foci, relative to control cells [64]. In combination, these results suggest that inefficient repair of DNA damage, through ESCO2-dependent defects in HR, is an underlying factor in RBS biology.

#### An ROS model for RBS

RBS birth defects may involve reactive oxygen species (ROS) pathways, a model in part supported by thalidomide teratogenicity. Thalidomide is a sedative and antimimetic that was used to treat morning sickness associated with pregnancy between 1957 and 1961. In utero exposure to thalidomide, however, induces severe birth defects, which resulted in its temporary removal from the market [81–83]. Due to an abundance of overlapping phenotypes, RBS was historically referred to as pseudothalidomide syndrome [3,6,84–86], but functional or mechanistic links between these 2 developmental maladies are only now emerging [87–90]. An important aspect of thalidomide teratogenicity is rooted in oxidative stress. Thalidomide is converted in vivo to an oxidative metabolite, dihydroxythalidomide (DHT), which is further oxidized to ROS-generating quinones that induce DNA damage [91]. In human embryonic kidney (HEK293) cells, DHT increases intracellular ROS, which in turn generates DNA damage in the form of double-strand breaks, as revealed through “Comet” assay analysis [92]. Importantly, DHT exposure is sufficient to produce developmental defects in rabbit embryos that include phocomelia [93], defects that are rescued by co-exposure to the ROS-neutralizing agent alpha-phenyl-N-t-butylnitrone [93]. This suggests that embryonic exposure to oxidative stress results in DNA damage that is, at least in part, a causative agent of phocomelia.

#### Macromolecular damage in RBS comes full circle

**Fig 2:**
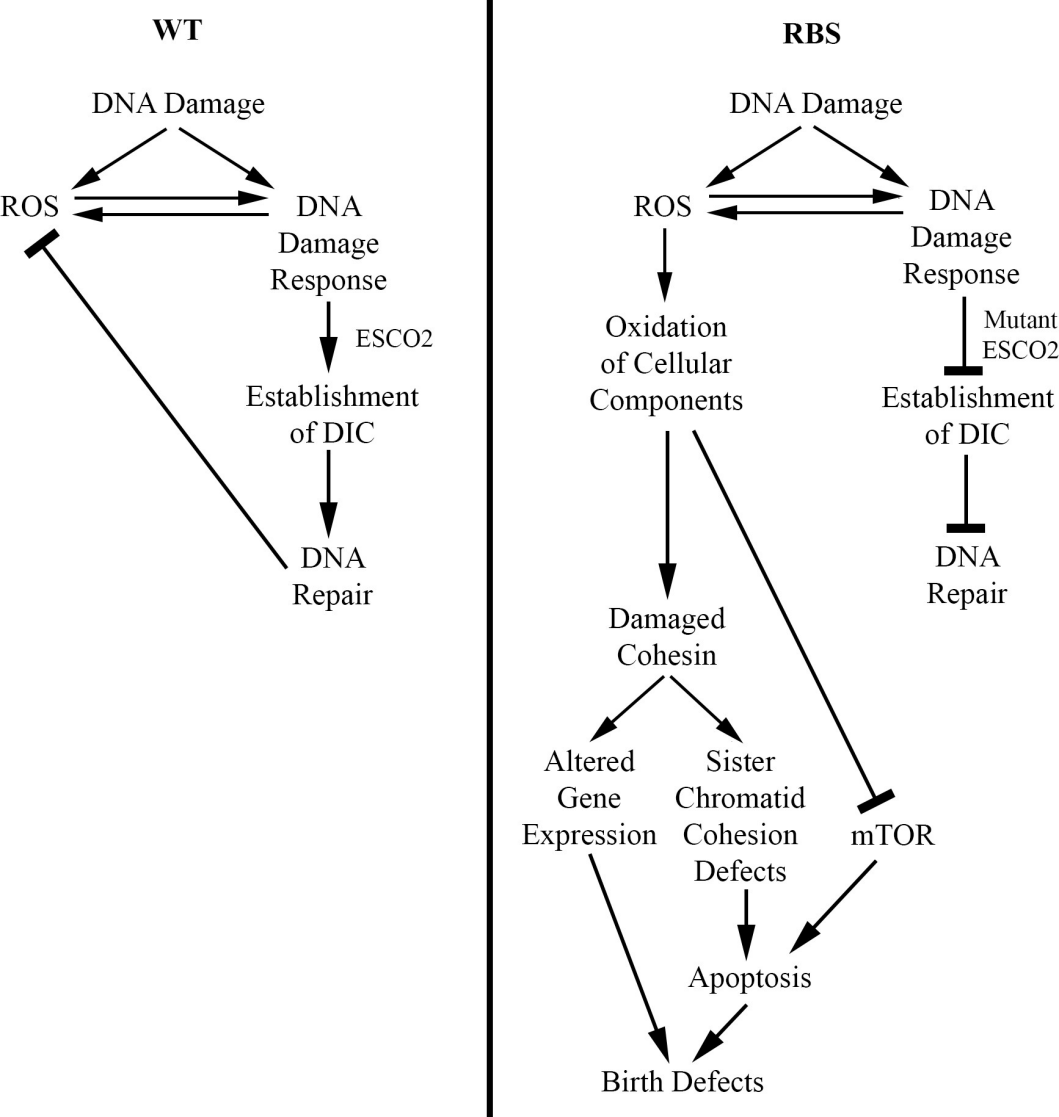
ESCO2 mutation increases ROS production. In normal cells (WT), DNA damage promotes ESCO2-dependent DIC and is efficiently repaired. DNA damage leads to transient up-regulation of ROS, which promotes DNA repair in combination with ESCO2 followed by reduction in ROS levels. In RBS, however, DNA damage leads to a dysregulated ROS positive feedback loop. Here, DNA damage up-regulates ROS, which is further induced by faulty DNA repair in the absence of proper ESCO2 function. Overproduction of ROS leads to oxidation of DNA and factors, which regulate the stress response, protein synthesis, and chromosome cohesion. The combination of reduced protein synthesis and elevated apoptosis results in RBS birth defects. DIC, damage-induced cohesion; ESCO2, establishment of cohesion 2; RBS, Roberts syndrome, ROS, reactive oxygen species; WT, wild type.

Oxidative stress is intimately linked to biological processes that are aberrant in RBS. For instance, unrepaired DNA damage up-regulates intracellular ROS to act as signaling molecules to promote stress responses [94–98]. Is it possible that *ESCO2* mutation is, in itself, sufficient to produce ROS? In fact, human RBS models and yeast cohesion mutants exhibit markers of increased ROS levels both in the absence of challenges and in response to DNA damage [63,74,97,99,100]. Mutual causality between DNA damage and ROS production may provide a synergistic mechanism, which enhances mutation rates in RBS cells. Evidence toward this end include yeast genetic interactions between HR regulators (including *ECO1*) and oxidative stress regulators that are associated with increased rates of spontaneous mutation and recombination [101–105]. In support of this, cohesin dysfunction is also a driver of ROS production and apoptosis. For example, mutation of *MCD1* (*RAD21*), and also *PDS5*, is sufficient to increase both ROS production and rates of apoptosis [99,100]. A possible consequence of a dysregulated ROS production loop may be elevated apoptosis rates—a hallmark of RBS cells (Fig 2) [10,33,37–39,94–98,106–116].

Cell death could be induced by a variety of non-mutually exclusive ROS-dependent mechanisms in RBS cells. For instance, ROS-dependent apoptosis funnels through oxidation of a variety of effectors including p53, the c-Jun N-terminal kinase (JNK) signaling kinase, the tumor necrosis factor (TNF)-α cytokine, and mitochondrial cytochrome *c*-release regulators [107–111,113,114]. Another potential mechanism through which DNA damage-induced ROS leads to apoptosis is oxidation of cohesin subunits. Evidence in support of this mechanism is that RNA interference (RNAi)-based KD of the ROS scavenger superoxide dismutase (SOD) in aged *Drosophila* oocytes increases chromosome arm cohesion defects and segregation errors that include nondisjunction [117]. Remarkably, overexpression of SOD in aged *Drosophila* oocytes reduces the frequency of sister chromatid nondisjunction [118]. This data raises the possibility that elevated rates of spontaneous DNA damage in RBS cells causes overproduction of proapoptotic ROS, which oxidizes cell death effector molecules and damages cohesion (Fig 2). Similarly, yeast cohesin mutants, exposed to ROS neutralizing agents, exhibit a reduced frequency of cell death [99], highlighting the likely connection between oxidative stress and apoptosis in RBS.

Separately, overproduction of ROS could compound complications that arise from reduced protein synthesis in RBS. Reduced ribosome function is associated with inhibition of mTOR signaling, which leads to increased apoptosis in a zebrafish model of RBS [63]. Additionally, translation is also inhibited during oxidative stress [119,120]. This indicates that elevated ROS levels could contribute to the RBS apoptotic phenotype due to its effect on translation and downstream inhibition of mTOR (Fig 2). Additionally, overproduction of ROS could affect gene transcription in RBS cells. ROS may directly oxidize DNA to effect transcription efficiency and further compound gene expression irregularities in RBS (Fig 2). Finally, ROS could affect gene expression by oxidation of cohesin proteins, thereby reducing cohesin-dependent transcription, which is already disrupted due to lack of ESCO2 function.

## Conclusions

The molecular etiology of RBS is complex and not yet fully understood. Pioneering work in uncovering the cellular basis for RBS identified both faulty chromosome cohesion and aberrant gene expression as playing critical roles in contributing to RBS birth defects. Defects in DNA repair, however, have long been associated with RBS cell biology—but a DNA damage component of RBS has gained little traction. DNA damage creates further oxidative damage in the cell which, in part, can explain known hallmarks of RBS cell biology including cohesion defects and dysregulated gene expression. Reciprocity may rule the day in that *ESCO2* mutations are sufficient to produce oxidative stress and ROS up-regulation. Mitigating the circular and self-enforcing effects of unrepaired DNA damage and ROS up-regulation in RBS individuals thus presents an exciting area of future research. This model may have far-ranging implications in that RBS is 1 member of a group of multi-spectrum developmental disorders that include Warsaw Breakage syndrome, Mungan syndrome, Mullegama–Klein–Martinez syndrome, Juberg-Hayward syndrome, epileptic encephalopathy, Baller–Gerold syndrome, and Cornelia de Lange Syndrome [9,10,18–23,48,64,121–123]. It may be of significant benefit to address the role of DNA damage and ROS up-regulation in these and other maladies (Diamond–Blackfan anemia, Treacher–Collins syndrome, and coloboma, heart defects, atresia choanae, retardation of growth, genital hypoplasia, and ear abnormalities (CHARGE) syndrome) implicated in cohesin mutation and that exhibit phenotypes that overlap with those of cohesinopathies [51,66,124,125].
